# Continued Endemic Wild Poliovirus Transmission in Security-Compromised Areas — Nigeria, 2016

**DOI:** 10.15585/mmwr.mm6607a2

**Published:** 2017-02-24

**Authors:** Chimeremma Nnadi, Eunice Damisa, Lisa Esapa, Fiona Braka, Ndadilnasiya Waziri, Anisur Siddique, Jaume Jorba, Gatei wa Nganda, Chima Ohuabunwo, Omotayo Bolu, Eric Wiesen, Usman Adamu

**Affiliations:** ^1^Global Immunization Division, CDC; ^2^National Polio Emergency Operations Center, National Primary Health Care Development Agency, Abuja Nigeria; ^3^Expanded Program on Immunization, World Health Organization, Nigeria Country Office; ^4^National Stop Transmission of Polio Program, Africa Field Epidemiology Network, Nigeria Country Office; United Nations Children’s Fund, Nigeria Country Office; ^5^Division of Viral Diseases, National Center for Immunization and Respiratory Diseases, CDC.

On August 10, 2016, 2 years after the most recent wild poliovirus (WPV) case was reported in Nigeria (in July 2014) (*1*), two WPV cases were reported in the northeastern state of Borno, which has been severely affected by insurgency-related insecurity since 2013. On September 9 and 26, 2016, two additional WPV cases were reported in Borno in children whose families migrated from security-compromised, inaccessible areas of the state. All four cases were WPV serotype 1 (WPV1), with genetic differences indicating prolonged undetected transmission. A large-scale emergency response plan was developed and implemented. The plan initially called for vaccination of 815,791 children during August 15–18 in five local government areas (LGAs) in the immediate vicinity of the first two WPV cases. Subsequently, the plan was expanded to regionally synchronized supplementary immunization activities (SIAs), conducted during August 27–December 6 in five Lake Chad basin countries at increased risk for national and regional WPV1 transmission (Cameroon, Central African Republic, Chad, Niger, and Nigeria). In addition, retrospective searches for missed cases of acute flaccid paralysis (AFP), enhanced environmental surveillance for polioviruses, and polio surveillance system reviews were conducted. Prolonged undetected WPV1 transmission in Borno State is a consequence of low population immunity and severe surveillance limitations associated with insurgency-related insecurity and highlights the risk for local and international WPV spread (*2*). Increasing polio vaccination coverage and implementing high-quality polio surveillance, especially among populations in newly secured and difficult-to-access areas in Borno and other Lake Chad basin areas are urgently needed.

## Security Situation

**FIGURE 1:**
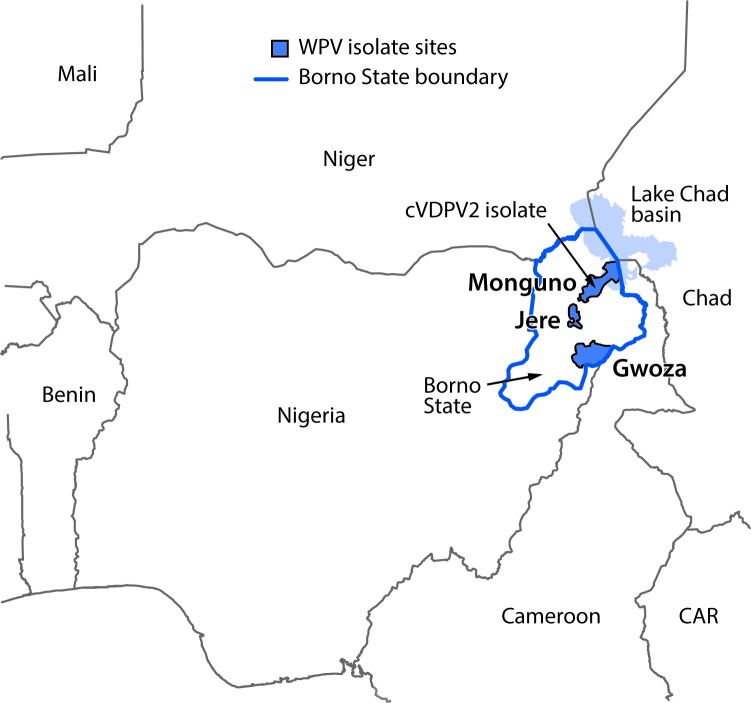
Location of wild poliovirus (WPV) isolates and circulating vaccine-derived type 2 poliovirus (cVDPV2) isolate identified in the local government areas of Jere, Gwoza, and Monguno — Borno State, Nigeria, 2016 **Abbreviation:** CAR = Central African Republic.

**FIGURE 2:**
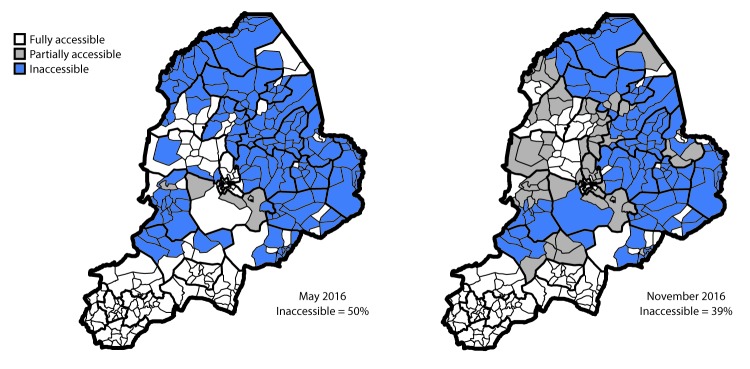
Security-related accessibility classifications within the 27 local government areas* — Borno State, Nigeria, May and November 2016 *Accessible population and settlement data for house-to-house and special vaccination teams.

Borno shares boundaries with Adamawa, Gombe, and Yobe states in Nigeria, and international boundaries with Cameroon, Chad, and Niger (Figure 1). Years of armed insurgency in Borno has led to destruction of the health care delivery infrastructure, including nearly two thirds of health facilities in the state. During the last 2 years, approximately half of all settlements in the state were inaccessible for implementation of effective polio eradication activities, including high-quality surveillance and immunization activities. An estimated 2.1 million internally displaced persons (IDPs) have sought shelter in formal and informal camp settings, as well as in communities in Borno and other Nigerian states (*3*). In addition, conflict-driven insecurity has led to the forced displacement of 200,000 refugees across international boundaries. Because of the prevailing humanitarian situation in Borno and other northeastern Nigeria states, in August 2016, the World Health Organization (WHO) declared a Grade 3 emergency in the region, indicating a substantial public health event requiring a major international response (*4,5*). Efforts by the Nigeria military have resulted in improved accessibility in Borno during the last year, although assessments in November 2016 indicate that 39% of settlements are still inaccessible because of insurgency-related insecurity (Figure 2).

## WPV Case Investigations and Response Plan

The first WPV case reported was in a child aged 23 months in Gwoza LGA, and the second was in a child aged 24 months from Jere LGA. Dates of paralysis onset were reported as July 4 and 13, 2016, respectively. The third and fourth cases were in two children aged 23 and 21 months from Monguno LGA, with respective dates of paralysis onset of August 6 and 21, 2016. Additional investigation identified an isolate of circulating vaccine-derived type 2 poliovirus (cVDPV2) in a healthy contact, aged 6 years, of one of the polio patients from Monguno LGA. cVDPVs are genetic variants of the oral vaccine virus that emerge and can cause paralysis indistinguishable from WPV disease in unimmunized or underimmunized populations (*6*). Laboratory analysis of the four WPV isolates showed limited genetic relationship among isolated viral strains; the closest known genetic link was to a virus last identified in Borno in 2013, indicating distinct and prolonged periods of undetected transmission (*7*). The cVDPV2 isolate was 37 nucleotides different from Sabin 2 and 25 nucleotides different from the closest match, also signifying prolonged undetected circulation. This was the second cVDPV2 isolate identified in Borno in 2016; the first isolate was from an environmental sample collected in March 2016 in Maiduguri LGA which had prompted SIAs with monovalent oral poliovirus vaccine type 2 (mOPV2) in May, June, and July (*2*).

In collaboration with Global Polio Eradication Initiative partners, health authorities in four other Lake Chad basin countries and staff members from the Nigeria Polio Emergency Operations Center planned and implemented a large-scale regional response during August–December 2016. The response included SIAs to vaccinate children against WPV1 and cVDPV2, intensified surveillance for AFP cases, and enhanced environmental surveillance (*8*). In addition, a review of polio surveillance activities in the region was conducted.

## Supplementary Immunization Activities

Following notification of the first two WPV cases, outbreak response vaccination using bivalent oral poliovirus vaccine (bOPV) (containing types 1 and 3 OPV) were conducted during August 15–18, 2016, in Jere and Gwoza LGAs and in three additional LGAs with substantial IDP populations. Five rounds of regionally synchronized SIAs also were implemented, targeting children aged <5 years in five Lake Chad basin countries (Cameroon, Central African Republic, Chad, Niger, and Nigeria) at risk for poliovirus transmission because of large population movements. SIA quality was evaluated using lot quality assurance sampling (LQAS) methodology. Overall, approximately 30 million children in 18 Northern Nigeria states were vaccinated with bOPV. In addition, one dose of inactivated polio vaccine (IPV) was administered to children in Borno State to boost immunity (Table). A separate outbreak response to the cVDPV2 detected in Monguno LGA was conducted using mOPV2 during December 2016 and January 2017.

**TABLE:** Polio outbreak response supplementary immunization activity dates, antigen types, target areas, number of children vaccinated, and reported lot quality assurance sampling (LQAS) results — Nigeria, August 2016–January 2017

Activity dates	Vaccine type	Target area	No. of children vaccinated	Percentage of LGAs achieving ≥90% on LQAS*
August 15–18, 2016	bOPV	Five Borno LGAs^†^	815,791	100
August 27–30, 2016	bOPV	Zone 1^§^	5,787,177	71
September, 17–20, 2016	bOPV	Zone 2^¶^	30,466,282	71
	IPV	Borno State	1,523,981	50
October 15–18, 2016	bOPV	Zone 2	31,422,237	86
November 12–15, 2016	bOPV	Zone 2	32,563,311	80
December 3–6, 2016	bOPV	Zone 2	32,449,576	85
December 16–19, 2016	mOPV-2	Zone 1 plus Bauchi and Sokoto states**	9,977,377	90
January 28–31, 2017	mOPV-2	Zone 2	Not available	Not available

**Abbreviations:** bOPV = bivalent (types 1 and 3) oral poliovirus vaccine; IPV = inactivated polio vaccine; LGA = local government area; mOPV-2 = monovalent (type 2) poliovirus vaccine.

* ≥90% coverage achievement pass mark on LQAS set by Nigeria polio Emergency Operations Center.

^†^ Immediate outbreak response in the Borno LGAs of Bama, Gwoza, Jere, and Maiduguri Municipal Council, and one accessible ward in Mafa.

^§^ Zone 1 = Adamawa, Borno, Gombe, Taraba, and Yobe states.

^¶^ Zone 2 = Abuja Federal Capital Territory, Adamawa, Bauchi, Borno, Gombe, Jigawa, Kaduna, Kano, Katsina, Kebbi, Kwara, Nasarawa, Niger, Plateau, Sokoto, Taraba, Yobe, and Zamfara states.

** Bauchi and Sokoto states were included as target areas because of increased risk profiles.

## Poliovirus Surveillance

**AFP surveillance.** In 2016, a total of 614 AFP cases were reported in Borno State, a 73% increase from 354 reported cases in 2015. Improvements in AFP reporting in 2016 can be attributed to increased surveillance activity in the wake of the March 2016 cVDPV isolation, as well as improving access to populations in previously inaccessible areas of the state.

**Environmental surveillance.** Following the reported WPV circulation in Borno, the number of environmental sampling sites in Borno was increased from four to six. In March 2016, the frequency of sample collection at existing sites in Maiduguri (the Borno State capital) was increased from monthly to weekly following the reported cVDPV2 isolation (*2*). No positive WPV or cVDPV isolate has been reported from any of the sites since April 2016.

**Surveillance reviews.** Two surveillance reviews were conducted as part of the response to assess and develop recommendations to improve weaknesses in surveillance that could account for the prolonged undetected transmission indicated by genetic sequence analyses of isolates from reported WPV1 cases. A key finding was the limited ability to conduct systematic high-quality surveillance in security-compromised areas of Borno State, which adversely affected case detection and reporting. In addition, serious surveillance performance limitations were identified, including inconsistent AFP case reporting in some fully accessible areas. Measures were recommended to strengthen surveillance activities at the national and subnational levels, including the development of protocols for improved identification of the location of cases among IDPs.

## Discussion

The recent finding of prolonged undetected WPV circulation in Borno State highlights key challenges facing polio eradication efforts in Nigeria and globally. Although the large-scale outbreak response SIA and surveillance activities conducted in the Lake Chad basin region were considered successful, conflict-related inaccessibility might continue to limit surveillance and immunization activities, raising concerns about further undetected WPV and cVDPV transmission. To reduce the potential for persistent virus transmission, it is important to increase polio surveillance quality and vaccination coverage among cohorts of persons in unimmunized and underimmunized populations, prioritizing persons living in recently accessible areas, IDP camps, and refugee communities.

As has been observed in Afghanistan and Pakistan, the two other countries that have not yet interrupted endemic WPV transmission, insurgency-related insecurity can restrict access to populations in conflict settings, potentially imposing limits on implementation of polio eradication activities, including high-quality immunization and surveillance activities (*9*,*10*). In Borno State, the restriction of the implementation of polio eradication activities in insurgent-held areas has been absolute: no polio eradication activities occurred in those areas. Despite multiple SIA rounds conducted in accessible areas of Borno State prior to August 2016 for which all children were eligible, two of the four children with WPV had never received polio vaccine, and the other two did not complete the polio vaccination series. This finding validates longstanding concerns about WPV circulation among populations that have become susceptible because of the inability to reach and fully vaccinate children in security-compromised areas. Although recent gains made by the Nigerian military have led to an increase in the number of areas now accessible to polio eradication activities, approximately 40% of settlement communities in Borno State are still classified as fully inaccessible. Unless a substantial proportion of children in these settlement communities are reached and vaccinated, it will be difficult to interrupt WPV transmission in the inaccessible areas of Borno State.

Large population migration to and from refugee and IDP camps and communities has occurred across Nigeria and other Lake Chad basin countries (*3*), increasing the potential for WPV transmission in settings far removed from the conflict. For this reason, immediate steps were taken to increase polio vaccination coverage in large areas of the Lake Chad region. The risk for outbreaks among IDPs in camps and host communities across the region remains, because of the continued migration of potentially infected and underimmunized persons from the security-compromised areas of Borno State.

Armed conflict limits the implementation of high-quality surveillance activities. The current polio surveillance system in Borno State has identified WPV cases, but exclusively in accessible areas of the state. The continued insurgency-related access limitation in a number of subdistricts in Borno State means that the risk for undetected transmission in these communities might persist until secure access becomes feasible. In addition, the recent findings of prolonged undetected WPV circulation in Borno State, along with other deficiencies highlighted in recent surveillance reviews, underscore the need for improved surveillance in areas that have become accessible. Regular and rigorous supervision, evaluations, and reviews focused on surveillance performance at the subdistrict level are urgently needed.

**Summary**

**What is already known about this topic?**

In August 2015, 1 year after the last known case of wild poliovirus (WPV) infection was reported in Nigeria in July 2014, the World Health Organization removed Nigeria from the list of endemic countries because of the high likelihood that endemic WPV circulation had been interrupted in Nigeria. However, Borno State in northeastern Nigeria has experienced years of armed insurgency, which has hampered implementation of effective polio eradication activities.

**What is added by this report?**

During August and September 2016, four WPV cases and one circulating vaccine-derived poliovirus (cVDPV) isolate were reported in accessible areas of Borno State. Analysis of the WPV isolates showed limited genetic relationship, indicating prolonged undetected transmission. In response, regionally synchronized supplementary immunization activities were conducted in five Lake Chad basin countries, and >30 million children in 18 northern states in Nigeria were vaccinated. Additional measures to strengthen polio surveillance quality were implemented in accessible areas of Borno State. Ongoing conflict-related insecurity continues to restrict polio workers’ access to populations in insurgent-held areas.

**What are the implications for public health practice?**

Although the areas that are insurgent-held have diminished over the last year, about 40% of communities in Borno State remain inaccessible. Response to the detection of WPV and cVDPV was highly successful in accessible areas. Increasing polio vaccination coverage and improving surveillance quality among cohorts of unimmunized and underimmunized populations is a critical public health need in Borno and the Lake Chad region.
